# Ecological Niche Modeling to Calculate Ideal Sites to Introduce a Natural Enemy: The Case of *Apanteles opuntiarum* (Hymenoptera: Braconidae) to Control *Cactoblastis cactorum* (Lepidoptera: Pyralidae) in North America

**DOI:** 10.3390/insects11070454

**Published:** 2020-07-19

**Authors:** Nidia Bélgica Pérez-De la O, Saúl Espinosa-Zaragoza, Víctor López-Martínez, Stephen D. Hight, Laura Varone

**Affiliations:** 1 Facultad de Ciencias Agrícolas, Universidad Autónoma de Chiapas, Entronque Carretera Costera y Estación Huehuetán, Huehuetán 30660, Chiapas, Mexico; belgica_delao@hotmail.com (N.B.P.-D.l.O.); saulez1@gmail.com (S.E.-Z.); 2 Facultad de Ciencias Agropecuarias, Universidad Autónoma del Estado de Morelos, Av. Universidad 1001, Col. Chamilpa, Cuernavaca 62209, Morelos, Mexico; 3 USDA-ARS, Center for Medical, Agricultural and Veterinary Entomology, 6383 Mahan Drive, Tallahassee, FL 32308, USA; stephen.hight@usda.gov; 4 Fundación para el Estudio de Especies Invasivas, Bolívar 1559, Hurlingham, Buenos Aires 1686, Argentina; lauvarone@fuedei.org

**Keywords:** invasive species, classical biological control, environmental suitability

## Abstract

The cactus moth, *Cactoblastis cactorum* (Berg) (Lepidoptera: Pyralidae), is an invasive species in North America where it threatens *Opuntia* native populations. The insect is expanding its distribution along the United States Gulf Coast. In the search for alternative strategies to reduce its impact, the introduction of a natural enemy, *Apanteles opuntiarum* Martínez and Berta (Hymenoptera: Braconidae), is being pursued as a biological control option. To identify promising areas to intentionally introduce *A. opuntiarum* for the control of *C. cactorum*, we estimated the overlap of fundamental ecological niches of the two species to predict their common geographic distributions using the BAM diagram. Models were based on native distributional data for both species, 19 bioclimatic variables, and the Maxent algorithm to calculate the environmental suitability of both species in North America. The environmental suitability of *C. cactorum* in North America was projected from Florida to Texas (United States) along the Gulf coastal areas, reaching Mexico in northern regions. *Apanteles opuntiarum* environmental suitability showed a substantial similarity with the calculations for *C. cactorum* in the United States. Intentional introductions of *A. opuntiarum* in the actual distribution areas of the cactus moth are predicted to be successful; *A. opuntiarum* will find its host in an environment conducive to its survival and dispersal.

## 1. Introduction

Mexico is one of the most rich and diverse areas of Cactaceae, with 850 species [1], many of them endemic to the region. Of the Cactaceae, the most widespread and common genus is *Opuntia*, naturally occurring from near the Canadian Arctic Circle to the tip of Patagonia in South America [2]. *Opuntia* spp., commonly referred to as prickly pear cacti, have long been used by native peoples as food for humans and animals, medicines, pigments, and fencing [3]. The most widely used and economically important species of *Opuntia* for food is *O. ficus-indica* (L.) Mill., selected by indigenous people in central Mexico long ago as a vegetable (nopalitos) and a fruit (tunas) [4]. Many other edible species are used in Mexico, including *O. megacantha* Salm-Dyck, *O. stricta* Haw., *O. dillenii* (Ker Gawl.) Haw., *O. schumannii* Weber, *O. robusta* Wendl., and *O. albicarpa* Scheinvar [5,6]. Mexico leads the world in prickly pear cactus food production (74%) and is also the principal consumer. Demand for nopalitos is increasing in foreign markets, principally the United States and Canada [4]. Mexico grows nopalitos on 12,853 hectares [7], with a food supply chain that supports many families across the country. Additionally, the cultivated area of cactus fruits grew 940% from 1980 to 2015 [8].

Insect pests are a strong biotic factor that limits *Opuntia* yield and quality [9], and some invasive herbivore species are considered a severe threat to this crop. One example of an invasive threat is the cactus moth from South America, *Cactoblastis cactorum* (Berg) (Lepidoptera: Pyralidae) [10]. Larvae of *C. cactorum* feed gregariously inside cladodes, causing the decay and often death of infested plants [11,12]. This South American species [12] was introduced intentionally into Australia and South Africa as a biological control agent that successfully controlled exotic *Opuntia* species [11,13]. Unfortunately, *C. cactorum* was introduced into the Caribbean for the control of invasive and native *Opuntia* species [14], and the moth spread naturally and by human-assisted introductions throughout the Caribbean [15]. *Cactoblastis cactorum* was found in the Florida Keys in 1989 [16] and has spread throughout most of the Florida peninsula, along the Atlantic Coast to North Carolina, and the Gulf Coast to Texas [17]. Outbreaks of *C. cactorum* populations occurred in Quintana Roo, Mexico, on Isla Mujeres in 2006 and Isla Contoy in 2007, but were eradicated by 2009 [18]. Because of its rapid geographical expansion and its reputation as a voracious *Opuntia* feeder, *C. cactorum* continues to threaten Mexico’s *Opuntia* diversity and industry [19].

Many strategies for the control of cactus moth populations have been evaluated, including the insect sterile technique [20], pheromones for monitoring presence and occurrence of the moth [21], and insecticidal applications [22]. However, none of these control strategies prevented the moth from spreading. The use of exotic biological control agents has been considered as a management tactic [23,24], and one parasitoid looks to be a promising candidate, *Apanteles opuntiarum* Martínez and Berta (Hymenoptera: Braconidae), because of its restricted host range to the genus *Cactoblastis* in the parasitoid’s home range of Argentina [25,26,27]. *Apanteles opuntiarum* is a gregarious larval parasitoid of *C. cactorum*, and *A. opuntiarum* colonies are currently maintained in a Florida quarantine facility undergoing host specificity testing on North American non-target species [28]. However, the potential areas where this braconid wasp can become established after release in North America are still unknown, an important aspect essential to the success of the biological control agent.

Any non-native species intentionally introduced must have the capacity to establish, survive, and multiply in local biotic and abiotic elements [29,30]. Failure of biological control programs has been deeply influenced by abiotic factors, such as climatic factors [31]. A strategy developed to select appropriate sites for the natural enemy’s liberation based on prioritizing its environmental requirements could increase the success of biological control agents [32]. Ecological niche modeling is a tool widely used for estimating environmental suitability for organisms, building ecological models for successful prediction in specific geographic areas of interest [33,34,35,36]. In this study, we calculated the ecological niche model for *A. opuntiarum* and *C. cactorum* to define regions of environmental suitability and determine niche overlap for the purpose of predicting areas of successful establishment of this parasitoid in North America.

## 2. Materials and Methods

### 2.1. Insect Distributional Data in Native Range

Field collections in the native Argentine range of *C. cactorum* and *A*. *opuntiarum* (scanning latitudes of 23°53′ S to 40°48′ S; and longitudes of 58°38′ W to 66°10′ W) were conducted from August 2007 to March 2014. Insect collection sites were selected along roadsides when *Opuntia* patches were identified. Sites with single and multiple *Opuntia* species were selected, and each *Opuntia* site was georeferenced. If the patch contained many *Opuntia* plants, 50 individuals of the same species were counted and visually inspected to detect cactus moth larval damage. For patches with fewer than 50 *Opuntia* plants, all individuals of the same species were examined. Plants exhibiting feeding damage were dissected and examined for larval detection. Late instar larvae were collected and reared individually on pads of the original *Opuntia* species and checked every 2–3 days for presence of parasitoid cocoons. A total of 394 sites and 495 cactus patches were studied. A database was created that recorded location information, *Opuntia* species, rearing information, and parasitoid outcomes. The total number of data positive for *C. cactorum* was 282 points (153 from field work, 15 from databases, and 114 from literature), and positive for *A. opuntiarum* was 145 (41 from field work and 105 from literature). A subset of these data were used to build robust ecological niche models [37] (see below).

### 2.2. Model Calibration 

The study (North America) and calibration (Argentina) areas for *A. opuntiarum* and *C. cactorum* were delimited using a correlative perspective [33]. Calibration area is defined as the region accessible via dispersal over relevant periods [37,38]. A niche for each species was constructed by correlating known insect distribution data with data sets on such factors as climate and ecological conditions (data in the form of GIS layers). Maxent algorithm methods were used to extrapolate associations between point occurrences of a species and the environmental data sets to identify areas of predicted presence on a map. Defining the appropriate geographic areas for modeling is a critical aspect for extrapolation algorithms because the modeled areas need to be ecologically similar to the area where the species live. A species distribution area was defined based on the “BAM diagram”, as outlined by Soberón and Peterson [33]. The BAM diagram takes into account three conditions that define a species’ presence: biotic factors, abiotic conditions, and species accessibility to areas in which to disperse [33]. Geographically, these areas were determined following the criteria of the terrestrial ecoregions of the world [39].

**Figure 1:**
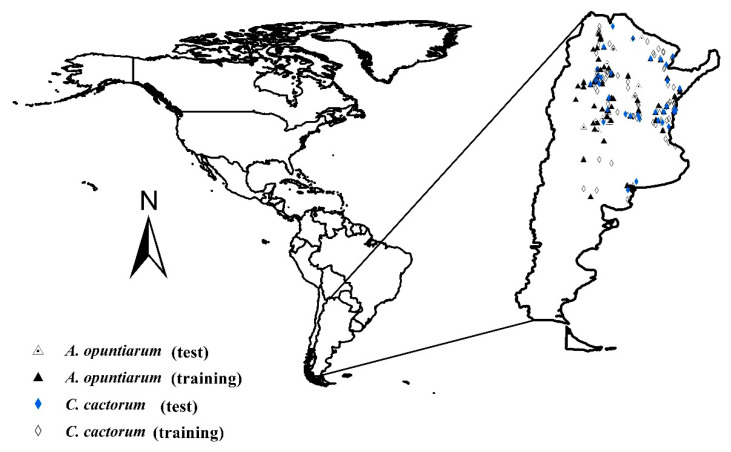
Occurrence data points recorded from the field and the literature for *Cactoblastis cactorum* and *Apanteles opuntiarum* in Argentina, used for ecological niche model construction.

To eliminate a potential spatial correlation, species occurrence points within a distance of 5 km or less from one another were removed from the analysis. The final database used to construct the ecological niche models included 101 points for training and 34 for testing the occurrence of *C. cactorum*, and 60 for training and 20 for testing *A. opuntiarum* occurrence (Figure 1). Information of 19 bioclimatic variables from WorldClim ver 2.0 (Sustainable Intensification Innovation Lab, Manhattan, NY, USA) was extracted from each point selected and a Spearman correlation in the Past 2.17c software was conducted to eliminate correlated bioclimatic variables. To compare statistical yield and explore results with a variable set with different correlation values, set 1 eliminated those variables with higher correlation values of 0.75 and −0.75 and set 2 used values of 0.9 and −0.9.

Candidate models were built with the R Kuenm [40] package and kuenm_calfunction in Rstudio^®^ ver. 3.3. (RStudio Team, Boston, MA, USA). The R Kuenm was used to develop three crucial stages in ecological niche modeling (ENM): model calibration, creation of candidate models, and an evaluation to determine the final model [40]. This package works with the modeling algorithm Maxent machine learning method with a simple and precise mathematical formulation [41]. For model construction, the Regularization multiplier was used to explore complex models and construct models with an increased robust regularization and to reduce overfit possibilities [42]. Maxent features were also used to construct the ENM by incorporating an expanded set of transformations of the original covariates [43]. Combinations of four values of Regularization multiplier (0.1, 0.5, 1, 2, and 3) and five Maxent features (Linear, Quadratic, Product, Threshold, and Hinge) produced a total of 580 models. Creating a range of different parameter sets is considered a more robust protocol to construct an ENM instead of using predetermined combinations [44].

### 2.3. Evaluation and Construction of Final Models

The accuracy of the models was evaluated with the area under the curve (AUC index) and classified under the following scale: 0.9–1 = excellent; 0.8–0.9 = good; 0.7–0.8 = fair; 0.6–0.7 = poor; and 0.5–0.6 = fail [45]. The 580 candidate models were evaluated with the R Kuenm package [40] and the function kuenm_cevalfunction. The evaluation metrics were the corrected Akaike information criteria (AICc) to penalize model complexity (models inappropriately complex or simple have the minor capacity to interfere in the habitat quality), Delta AICc (the probability that the model is the best, among the set of constructed models) [46], Partial Roc (graphs the proportion of correct occurrences within the predicted area at which the omission error is low enough to meet the predictive capacity requirements, α ≤ 0.05) [47], and the Omission rate (minimizes overfitting to calibration data; ≤%, with the kuenm_cevalfunction function) [48,49]. The evaluation points were randomly selected (25% of the field points and 25% of the literature points).

### 2.4. Extrapolation Risk Analysis and Selection of the Final Model

Considering that automatic learning algorithms such as Maxent maximize the model fit to the calibration data [48], we selected the most parsimonious model by choosing those with the best combination of Partial Roc (values near zero meant that all evaluation points were within the predicted area), Omission rate (fewest proportion of unpredicted species presence) [49], AICc (lowest value represented the best model), Delta AICc (values under 2 empirically supported the candidate model), and number of parameters (the least number of parameters equaled the simplest answer) [40,50]. When working with an invasive pest, a model is sought that does not leave out areas that could be suitable for the species. The characteristics of the selected model for *C. cactorum* were: Partial Roc: 0, Omission rate: 0, AICc: 3997.29, Delta AICc: 0, and the number of parameters was 18. The characteristics for the *A*. *opuntiarum* model were: Partial Roc: 0, Omission rate: 0.15, AICc: 2257.0144, Delta AICc: 1.366, and the number of parameters was 7.

Characterizing new or different environments becomes important when transferring models, so several steps have been followed to identify the model’s response to new environments with a novel combination of variables [38]. Mess analysis measures the similarity of the calibration area with the extrapolation area by assigning negative values to cells with different environments and positive values to the most similar. In this way, it is possible to identify regions where the predictions (similarity/dissimilarity) need to be evaluated [51]. Analysis of environmental suitability was based on political division maps.

## 3. Results

### 3.1. Environmental Suitability of Cactoblastis cactorum in North America

**Figure 2:**
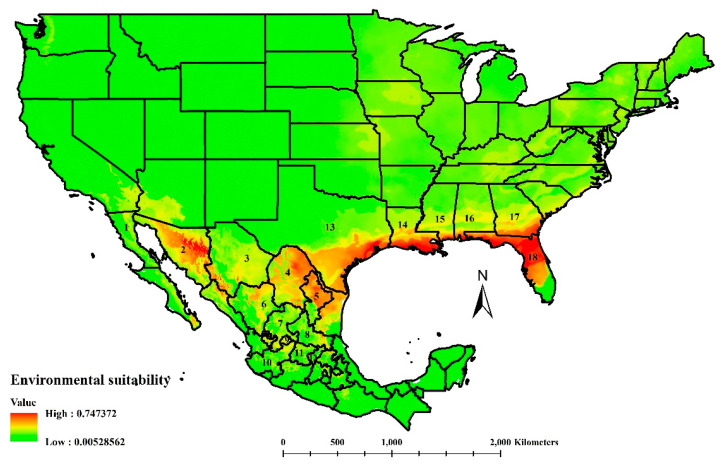
Environmental suitability for the cactus moth, *Cactoblastis cactorum*, in North America according to the division by states for Mexico and the United States. State identifications for Mexico include (1) Baja California, (2) Sonora, (3) Chihuahua, (4) Coahuila, (5) Nuevo Leon, (6) Durango, (7) Zacatecas, (8) San Luis Potosi, (9) Aguascalientes, (10) Jalisco, (11) Guanajuato, and (12) Queretaro. State identifications for the United States includes (13) Texas, (14) Louisiana, (15) Mississippi, (16) Alabama, (17) Georgia, and (18) Florida. As the environmental suitability level moved towards the red end of the scale, a more significant environmental suitability for *C. cactorum* was calculated.

The model selected for *C. cactorum* produced an AUC value of 0.811, revealing excellent predictive occurrence performance. Five bioclimatic variables determined the environmental suitability of *C. cactorum* in Mexico and explained 99.9% of the variation within the model. These bioclimatic variables were the mean temperature of the coldest yearly quarter (52.9%), temperature seasonality (33.2%), temperature annual range (7.4%), precipitation during the warmest quarter (4.4%), and precipitation during the coldest quarter (2.0%). The environmental suitability calculated for *C. cactorum* in North America was restricted to the southeastern United States; running from Florida to Texas, entering deeply into Mexico and covering almost entirely Coahuila, Nuevo Leon, and Tamaulipas, with projected suitability in Durango, Chihuahua, and Sonora (Figure 2). Medium environmental suitability calculation occurred irregularly across central Mexico and reached the southernmost areas in Oaxaca. In the United States, *C. cactorum* suitability was projected to have an affinity for the Gulf Coast (Figure 2).

Locations of Mexican edible prickly pear cactus farms [7] where the *C. cactorum* niche coincides occurred mainly in Aguascalientes, Baja California, Baja California Sur, Coahuila, Durango, and Zacatecas (Figure 2). Minor cactus production areas that could be suitable for this moth are found in Mexico City, the state of de Mexico, Guanajuato, Jalisco, Nuevo Leon, Queretaro, Sonora, and Tamaulipas. A minimal area of suitability was calculated to occur in Oaxaca. However, the principal production area of edible prickly pear in Morelos did not show environmental suitability for *C. cactorum*.

### 3.2. Environmental Suitability for Apanteles opuntiarum in North America

The model selected for *A. opuntiarum* produced an AUC value of 0.857, an excellent predictor of environmental suitability for this species. Eight bioclimatic variables determined the environmental suitability of *A. opuntiarum* in Mexico and explained 100% of the variation within the model. The variables were mean temperature of the coldest yearly quarter (34.4%), precipitation during the warmest quarter (26.9%), precipitation seasonality (17.3%), isothermality (13.4%), precipitation during the driest quarter (3.8%), annual precipitation (2.5%), precipitation during the coldest quarter (1.4%), and temperature seasonality (0.3%).

**Figure 3:**
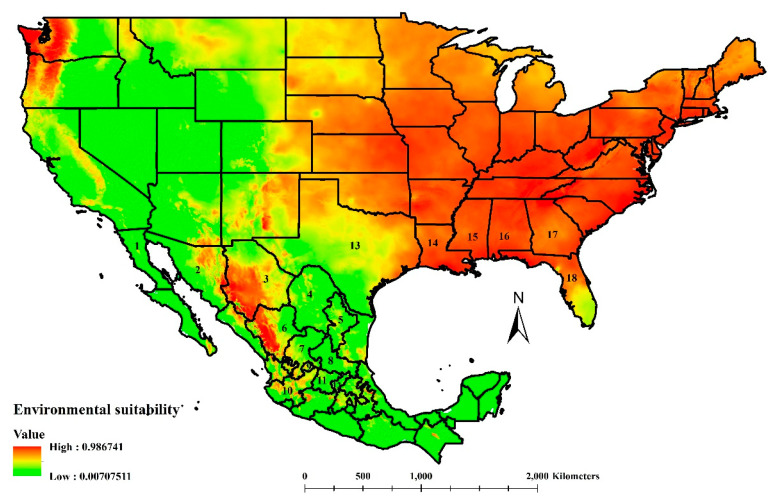
Environmental suitability for *Apanteles opuntiarum*, a parasitoid of *Cactoblastis cactorum* in North America according to the division by states for Mexico and the United States. State identifications for Mexico includes (1) Baja California, (2) Sonora, (3) Chihuahua, (4) Coahuila, (5) Nuevo Leon, (6) Durango, (7) Zacatecas, (8) San Luis Potosi, (9) Aguascalientes, (10) Jalisco, (11) Guanajuato, and (12) Queretaro. State identifications for the United States includes (13) Texas, (14) Louisiana, (15) Mississippi, (16) Alabama, (17) Georgia, and (18) Florida. As the environmental suitability level moved towards the red end of the scale, a more significant environmental suitability for *A. opuntiarum* was calculated.

The environmental suitability for *A. opuntiarum* was different than the calculated areas for *C. cactorum* (Figure 3). In the United States, the model showed broad suitability for the eastern half of the country and a potential match in Oregon and Washington states. Medium environmental suitability was projected in central-northern Montana and northern Idaho. The parasitoids’ highest suitability ran from Florida to Texas, with irregular medial to lower suitability areas across New Mexico, Arizona, and California. In Mexico, the potential areas of suitability continued from Arizona and New Mexico and expanded into Sonora, Chihuahua, Durango, and northern Sinaloa. Medium suitability was calculated from Zacatecas, Nayarit, Jalisco, Michoacan, state of Mexico, and many irregular suitability patches projected in Coahuila, Nuevo Leon, Tamaulipas, San Luis Potosi, Veracruz, Puebla, and reaching Chiapas (Figure 3). *Apanteles opuntiarum* suitability coincided with a significant area of edible prickly pear cactus grown in the following Mexican states [7]: Aguascalientes, Baja California, Baja California Sur, Guanajuato, Jalisco, Queretaro, San Luis Potosi, and Sonora (Figure 3).

### 3.3. The Intersection of Cactoblastis cactorum and Apanteles opuntiarum Ecological Niches

**Figure 4:**
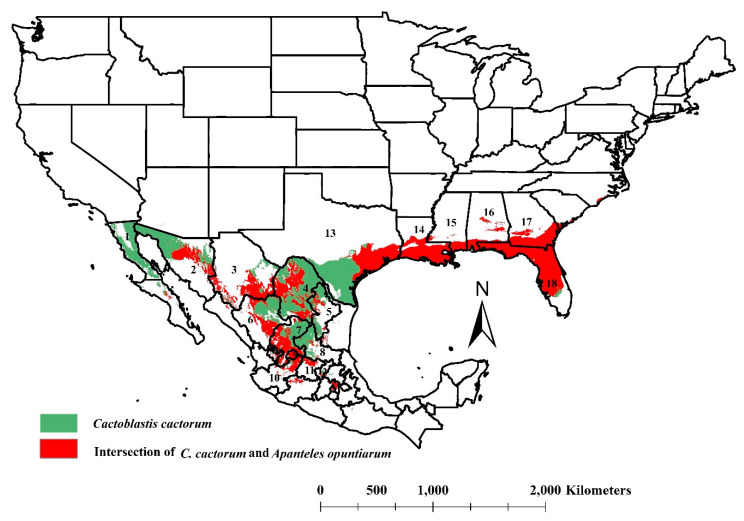
Intersection of ecological niches for *Cactoblastis cactorum* and its natural enemy *Apanteles opuntiarum* (red color) in North America. Areas in green are the ecological niche of the *C. cactorum* that would not intersect with *A. opuntiarum*. State designations for Mexico includes (1) Baja California, (2) Sonora, (3) Chihuahua, (4) Coahuila, (5) Nuevo Leon, (6) Durango, (7) Zacatecas, (8) San Luis Potosi, (9) Aguascalientes, (10) Jalisco, (11) Guanajuato, and (12) Queretaro. State designations for the United States includes (13) Texas, (14) Louisiana, (15) Mississippi, (16) Alabama, (17) Georgia, and (18) Florida.

The *A. opuntiarum* niches projected in the United States coincided with practically all the environmental suitability regions calculated for *C. cactorum* (from Georgia to Texas), except for small areas in southern Florida and middle southern Texas. In Mexico, the coincident pattern was irregular, with small patches in Central Baja California, Central Sonora, southern and southwestern Chihuahua, central and northern Nuevo Leon, western Tamaulipas, central and northern Durango, central-southern Zacatecas, and all of Aguascalientes (Figure 4). Small irregular patches were projected in San Luis Potosi, Jalisco, Michoacan, State of Mexico, Mexico City. For cactus growers, this interaction was calculated in Sonora, Coahuila, Tamaulipas, Zacatecas, Durango, State of Mexico, and Mexico City [7] (Figure 4).

### 3.4. Mess Analysis

**Figure 5:**
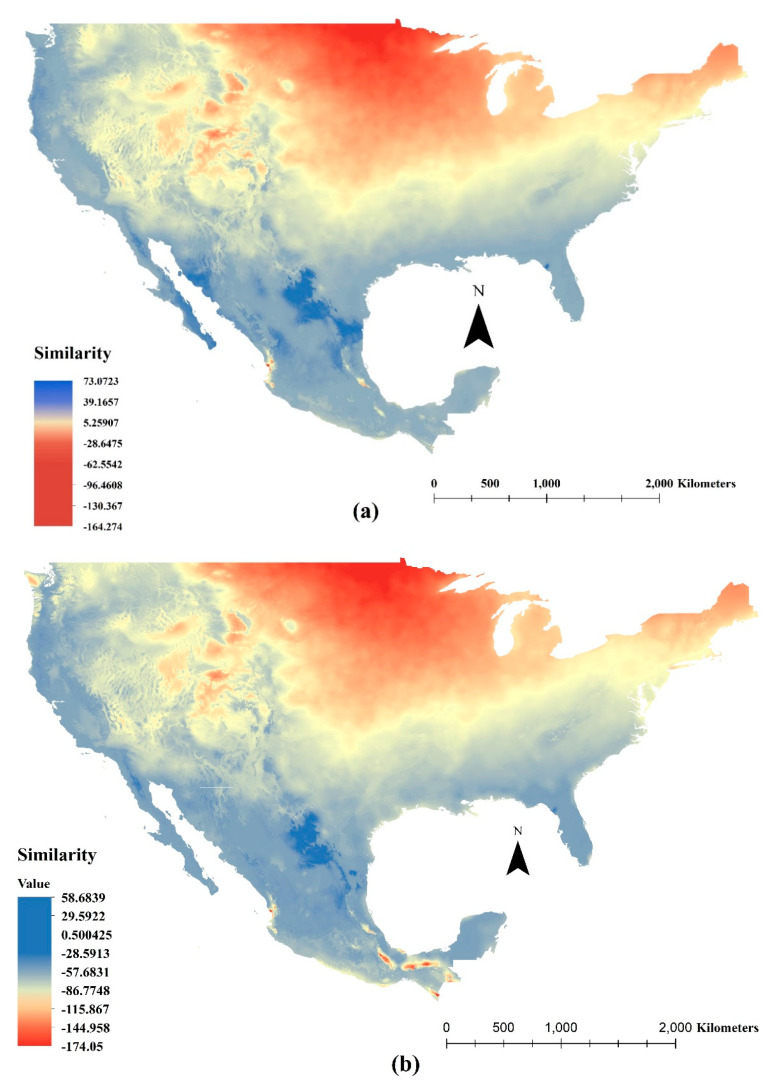
Mess analysis in North America for (**a**) *Cactoblastis cactorum*, (**b**) *Apanteles opuntiarum.* Scale bar shows the analogous level between the calibration (Argentina) and the transference areas (North America). Dark blue color and positive values indicate sites with climatic conditions similar to those in the insect species’ native ecological niche; dark red color and negative values indicate degrees of dissimilarity and that at least one bioclimatic variable was outside the range of the insect species’ native niche [52].

The five bioclimatic variables used for the Mess analysis to identify similar climatic areas for *C. cactorum* between Argentina and North America were the same variables identified as important in the development of the environmental suitability model for this species (see Section 3.1). Mess analysis to identify climate similarity for *C. cactorum* changed between countries (Figure 5a). The United States showed climate similarity in areas from the east to the west; from the Mid-Atlantic region along the Gulf of Mexico, and along the Pacific coastal areas. In the central to northern areas of the United States and high elevation areas in the Rocky Mountains, the similarity in climate diminished gradually to its minimal value. The similarity in climate areas for Mexico occurred in the northeastern and northwestern regions of the country, with dissimilarity in central Pacific areas and parallel areas to the Gulf of Mexico. Minor dissimilar areas were defined in the border with Guatemala.

The eight bioclimatic variables used for the Mess analysis of *A. opuntiarum* were the same variables identified as important in the development of the environmental suitability model of this species (see Section 3.2). Mess analysis identified *A. opuntiarum* climate similarity areas that resembled the results calculated for *C. cactorum* (Figure 5b). Areas with climate similarity in the United States and Mexico for *A. opuntiarum* were virtually identical to that projected for *C. cactorum*. In Mexico, however, areas with climate dissimilarity were projected as an irregular band reaching the Guatemalan border.

## 4. Discussion

Adequate prediction of susceptible areas where invasive species will establish is a useful tool in ecological studies and for designing strategies to reduce their negative impacts [53]. Our ecological niche modeling study with *C. cactorum* and *A. opuntiarum* estimated the geographic space that could be occupied by these two species in a new region. For the spotted lanternfly, *Lycorma delicatula* (White), an Asiatic species, using this methodology made it possible to calculate the ecological niche projected for *L. delicatula* in several fruit growing states in the United States, serving as a tool for design sampling and control strategies [54].

*Cactoblastis cactorum* and *A. opuntiarum* shared four bioclimatic variables to calculate their environmental suitability: mean temperature of the coldest quarter, temperature seasonality, precipitation during the warmest quarter, and precipitation during the coldest quarter. For both species, the essential bioclimatic variable was mean temperature of the coldest quarter, an index that provides mean temperatures during the coldest three months of the year and can be useful for examining how such environmental factors may affect species seasonal distributions [55]. In fact, lower temperatures are recorded as a limiting factor for *C. cactorum* eggs hatching, with probable lower and upper temperature thresholds for egg development of 20 and 30 °C, respectively [56]. For larvae, survival rate increased with temperature, with a lower temperature threshold of 18 °C [57]. For microgastrinae braconid wasps, temperature is a limiting factor for larval survival and even affects parasitoids emergence for synchronizing with their host [58]. However, for *A. opuntiarum*, the number of bioclimatic variables (8) involved in defining their ecological niche could indicate a more complex effect of climate in their survival.

We considered our projection of the *C. cactorum* ecological niche calculated in this study (Figure 2) to be adequate but improvable. The cactus moth invaded the continental United States through the Florida Keys [16] by anthropogenic activities [58,59] or natural phenomena [60]. The insect expanded its invasion range in the United States following an expansion pattern westward along the Gulf Coast and northward along the Atlantic coast [16,60,61]. Occurrence of *C. cactorum* was reflected in our modeled projection calculated here, except for the southernmost area in Florida where *C. cactorum* is reported to occur [16,61]. This dissimilarity could be an effect of not having sufficient data to represent the real ecological niche of the cactus moth in the native range distribution. However, our model successfully pointed out the actual dispersion pattern of *C. cactorum* in the United States.

The successful establishment and spread of invasive species, particularly those with some level of host specialization such as *C. cactorum*, is based on finding suitable food sources in the new environment [59]. Sufficient levels of cactus moth host plants in the genera *Opuntia* are common across the area currently invaded by the cactus moth [59,60,61]. A more-or-less continuous occurrence of *Opuntia* host plants along the Gulf of Mexico Coast region is a favorable ecological factor for continued dispersion of *C. cactorum* across this area and a high potential invasion risk for Mexico.

The federal states with environmental suitability for *C. cactorum* calculated in northern Mexico are considered a part of the arid subtropical region [62]. In this area, invasion by *C. cactorum* represents a threat to non-cultivated and cultivated *Opuntia*. Species of *Opuntia* grow in arid and semiarid areas of the country with at least 104 species recognized in Mexico; 56 are in the subgenus *Platyopuntia* (prickly pears) and 38 are endemics [63]. Additionally, northern Mexico is considered a radiation center for *Opuntia* [64]. From an economic point of view, the potential damage to edible cactus represents a profound impact on this food source used for human consumption and fodder for animals. *Opuntia* species have been a traditional food in Mexico for thousands of years [65] and comprise one of the most important natural resources for peasants and farmers in Mexico [3]. Farmers from at least 11 states (Aguascalientes, Baja California, Coahuila, Durango, Guanajuato, Jalisco, Nuevo Leon, Queretaro, San Luis Potosi, Sonora, and Zacatecas) will be forced to control the cactus moth as it invades Mexico. Traditionally, edible cactus farmers employ organophosphates to control pest populations [66], which have severe collateral damage to the environment, workers, and consumers. For Mexico, the invasion of *C. cactorum* will add to the list of invasive fruit destructive insect pests, such as *Diaphorina citri Kuwayama (Hemiptera: Liviidae),* a vector of the citrus huanglongbing disease [67], and the pink hibiscus mealybug, *Maconellicoccus hirsutus* (Green) (Hemiptera: Pseudoccocidae), a severe pest for ataulfo mango [68]. Both invasive insect species spread quickly, attack several hosts, and invade many regions in the country [69,70].

A successful biological control program is characterized by natural enemy survival and dispersion from the introduction site. For *A*. *opuntiarum*, their environmental suitability matched efficiently in the currently invaded areas of *C*. *cactorum* in the United States and even expanded to reach the central coastal areas of Texas where *C*. *cactorum* recently invaded. Our niche modeling presents an excellent opportunity to properly choose *A. opuntiarum* liberation sites in the United States with suitable ecological factors to ensure the parasitoids’ survival and dispersal. The same site selection criteria could be expected in an *A. opuntiarum* liberation effort in Mexico. In both countries, sites with suitable environmental conditions for *A*. *opuntiarum* survival were identified, increasing the likelihood for the parasitoid to be in contact with cactus moth larvae, increasing the chances for their survival and persistence [32].

The selection of an appropriate model in a robust and accurate ecological niche modeling study is essential to provide reliable data for policy-making authorities [54,71]. Accurate models could help in solving economic and human resource issues and shortcomings by concentrating efforts on the most appropriately selected sites. Our model supported the biological control option for reuniting the pest *C. cactorum* with its parasitoid *A. opuntiarum*, because the ecological niches for pest and natural enemy matched. Other ecological niche modeling studies have benefited biological control by determining that niche overlap between pest and the natural enemy did not match. For example, when ecological niches were calculated for two potential South American biological control agents, *Cochylis campuloclinium* Brown (Tortricidae) and *Liothrips tractabilis* Mound and Pereyra (Thripidae), under consideration for introduction into South Africa for control of the invasive pompom weed, *Campuloclinium macrocephalum (Less.) DC (Compositae)*, poor niche overlap was calculated for their environmental suitability and the agents were not pursued for introduction [72].

## 5. Conclusions

Ecological niche models were created for the invasive cactus moth *C. cactorum* and its parasitoid *A. opuntiarum* using GIS occurrence data in Argentina. The model confirmed the invasion pattern of *C. cactorum* in the United States and revealed a threat for Mexico from the Gulf Coast areas extending north to Coahuila, Nuevo Leon, and Tamaulipas states. The ecological niche model also calculated areas with environmental suitability for *A. opuntiarum* in the United States and determined areas that matched with the actual infested areas of *C. cactorum*, opening up the opportunity for planning liberation sites based on their predicted co-occurrence. If *C*. *cactorum* follows the modeled invasion pattern into Mexico, native and cultivated cactus species in suitable areas will face damage from the cactus moth. Releases of *A. opuntiarum* could be a useful tool when targeted in areas where its ecological niche shares environmental suitability with its lepidopteran pest.
